# DEAD-box RNA Helicase 39 Promotes Invasiveness and Chemoresistance of ER-positive Breast Cancer

**DOI:** 10.7150/jca.37247

**Published:** 2020-01-20

**Authors:** Xiudi Wang, Peipei Li, Chenying Wang, Dagui Zhang, Linghui Zeng, Xiyong Liu, Jiajin Lin

**Affiliations:** 1 The Second Affiliated Hospital and Yuying Children's Hospital of Wenzhou Medical University, Wenzhou, China 325027; 2 School of Medicine, Zhejiang University City College, Hangzhou, Zhejiang, China 310015; 3 Department of Tumor Biomarker Development, Sino-American Cancer Foundation, Covina, CA, USA 91722

**Keywords:** DEAD-box helicase 39 (DDX39), breast cancer, prognostic biomarker, chemoresistance, bioinformatics

## Abstract

**Purpose:** DDX39 is a DEAD-box RNA helicase that unwinds double-stranded RNA in an ATP-dependent manner. This study evaluated the prognostic and predictive significance of DDX39 in breast cancer (BC).

**Methods:** The cellular proliferation, invasion, and drug cytotoxicity by DDX39 siRNA were evaluated in MCF7 (ER-positive) and MDA-MB-231 (ER-negative) cell lines. A total of 27 datasets (total 8110 accessible cases) with following-up information were collected from Asia, Europe, and North America to explore associations between DDX39 gene expression and clinical parameters of BC patients.

**Results:** Down-regulation of DDX39 by siRNA significantly reduce the cell growth and invasion ability in MCF7 cells, but only slightly in MDA-MB-231 cells. The DDX39 mRNA level was elevated in breast adenocarcinoma compared with normal breast tissue (p<0.01). Higher DDX39 level was significantly correlated with larger tumor size (p<0.01) and poorer tumor differentiation (p<0.01). The prognostic significance of DDX39 for BC was assessed by pooled-analysis and meta-analysis. Kaplan-Meier analysis demonstrated that increased DDX39 mRNA expression was associated with poor outcomes significantly in a dose-dependent manner in ER-positive BC. The prognostic performance of DDX39 mRNA was comparable to 21-gene, 70-gene, and wound-response gene signatures, and it was superior to the TNM stage. Lower DDX39 expression was associated with reduced relative risk death on ER-positive BC with chemotherapy or radiotherapy. Inhibition of DDX39 by siRNA could significantly enhance the sensitivity of MCF-7 to doxorubicin.

**Conclusion:** DDX39 may be a potential novel prognostic and predictive biomarker for BC patients with ER-positive status.

## Introduction

DDX39 is an Asp-Glu-Ala-Asp (DEAD)-box RNA helicase that unwinds double-stranded RNA in an ATP-dependent manner 1. It plays roles in transcription, splicing, ribosome biogenesis, RNA export, RNA editing, RNA decay, translation, and telomere protection and maintenance 1-4. In recent years, DDX39 promotes tumor progression have been reported. DDX39 is up-regulated in lung squamous cell cancer and promotes cancer cell growth 5. DDX39 is a prognostic biomarker for gastrointestinal stromal tumor and hepatocellular carcinoma (HCC) 6 and promotes HCC migration, invasion, growth, and metastasis of cancer 7. However, the above findings could not be validated in a bladder cancer study. Overexpression of DDX39 inhibits the invasion of bladder cancer cells, and prognoses better outcome in bladder cancer 8. Although DDX39 was involved in the progression in many tumors, its role in breast cancer (BC) progression and regulatory mechanisms have not been investigated yet.

Globally, BC has been identified as the most frequent estrogen-related cancer type and the leading cause of cancer-associated mortality among women 9. Related studies have shown that up to 10% of women diagnosed with BC develop localized regional recurrence and up to 30% of distant metastases 10. However, approximately 90% of BC-related mortality is attributed to the formation of metastatic lesions 11, 12. Based on the specific genetic profile of estrogen receptor (ER), progesterone receptor (PR), and human epidermal growth factor-2 (Her2), BC patients can be classified into four categories: Luminal A, Luminal B, HER2-positive, and triple-negative subtypes 13, 14. Luminal A and luminal B BC have a high expression of the estrogen receptor (ER). HER2-positive and basal-like/triple-negative breast cancers (TNBCs) are ER-negative and are associated with a poorer prognosis 15. Knowledge of the subtype and accordingly the receptor status are required to decide the need for endocrine therapy and chemotherapy for a BC patient 16. ER-positive BC accounts for 60% ~ 70% of all BCs 17. The clinical guidelines have recommended the use of tamoxifen and aromatase inhibitors (AI) as adjuvant hormonal therapy options for women with ER-positive BC 18, 19. Most of ER-positive BC have a relatively good prognosis of tumor biology. However, few of them were at risk of relapse or metastasis after the primary resection, even post five years of treatment 20-22. Therefore, prognostic indicators are valuable for ER-positive BC to guide the physician for further therapeutic protocol selection.

Nowadays, a variety of multi-gene-based signatures have been developed to assess the risk of recurrence of ER-positive BC 23-26, such as the molecular-based risk of recurrences (ROR) 27, Oncotype DX (21-gene) recurrence score 26, breast cancer index (BCI) and EndoPredict (EP) 28. These detections provided useful information for the prediction of the outcome for ER-positive BC. On the other hand, these signatures provided a bulk of eligible target genes for target drug discovery. Nevertheless, these genes need to be further evaluated as a proper therapeutic target for novel anticancer drug discovery.

The present study addressed the hypothesis that DDX39 could prognosticate poor outcomes and might serve as a prognostic biomarker for BC. The roles of DDX39 on cell proliferation and invasion were investigated in ER-positive (MCF-7) and ER-negative (MDA-MB-231) BC cell lines. The prognostic significance of DDX39 was assessed on 27 independent gene expression datasets in individually and in a pooled manner. The association between DDX39 and chemoresistance was demonstrated in both the MCF7 cell line and validated on public gene expression datasets. Meanwhile, the protein-protein interaction network of DDX39 was also explored by bioinformatic analysis.

## Material and Methods

### Cell culture

MCF-7 (ER-positive), ZR-75 (ER-positive), and MDA-MB-231 (ER-negative) BC cell lines were obtained from Stem Cell Bank of the Chinese Academy of Sciences. Frozen aliquots were stored in the liquid nitrogen vapor phase, and cells were cultured for less than six months after thawing. These ATCC cell lines were authenticated before experiments. Cells were cultured in Dulbecco's modification of Eagle's medium (Hyclone, Logan, UT) supplemented with 10% fetal bovine serum (Bovogen, Essendon, Australia) and penicillin and streptomycin (Genom, Zhejiang, China). Doxorubicin (or Adriamycin) was obtained from Selleck Chemicals (Houston, TX, USA).

### Preparation of siRNAs and transfection

Synthesized siRNA duplexes were obtained from GenePharma Company (GenePharma Co., Ltd, Shanghai, China). The siRNA sequence is targeting DDX39 corresponded to nucleotides 185-203 (5'-GCAGCAGUACUACGUCAAATT-3', si-DDX39#1), and 162-180 (5'-GCACCAAAGGCCUAGCCAUTT-3', si-DDX39#2) of the coding region. The sequence for the negative control (si-NS) was 5'-UUCUCCGAACGUACGUTT-3'. BLAST searches were further performed to ensure that the sequence would not target other gene transcripts in the human genome database.

Seeded cells were incubated overnight, then transfected with siRNA (si-NS or si-DDX39) using siRNA-mate (GenePharma Co., Ltd, Shanghai, China), according to the manufacturer's protocol. The final concentration of siRNA was 50 nM. Silencing was examined 24 h after transfection. Total RNA was extracted from cell lysate by using TRIzol (Ambion) according to the manufacturer's instructions. 1 μg total RNA from each sample was added to the reverse transcription system.

### Western blot analysis

Briefly, cells were lysed in RIPA buffer (150 mM sodium chloride, 1% NP-40, 0.5% sodium deoxycholate, 0.1% sodium dodecyl sulfate and 50 mM Tris, pH 8.0), and protein was quantified by BCA assay (Beyotime Institute of Biotechnology). Proteins separated on 10% polyacrylamide gel were transferred to polyvinylidene difluoride membranes (Millipore, Billerica, MA) and detected using antibodies to actin (Beyotime Institute of Biotechnology), DDX39 (Abcam), ERK, AKT, p-ERK, p-AKT, E-cadherin and Vimentin (Cell Signaling Technology).

### *In vitro* invasion assay

The invasion ability of cancer cells is described as the movement of cells through extracellular matrices. Details of the invasion assay are described in our previous publication 29. About 20000 cells were seeded on the Matrigel™ (BD Company) insert of the 24-well chamber. After incubation for 24 hours, cells that had not migrated through the membrane in the Matrigel™ inserts were removed by a cotton-tipped swab. The invasion cells were stained with crystal violet staining solution (Beyotime Institute of Biotechnology) and counted. Each experiment was performed three times.

### *In vitro* cell proliferation and cytotoxicity assay

Cell Counting Kit-8 (CCK8; Beyotime Institute of Biotechnology, Jiangsu, China) was used to determine the number of viable cells in proliferation and cytotoxicity assays. Cytotoxicity, the quality of being toxic to cells, was quantified by a decrease in viable cell number after exposure to reagents. According to the manufacturer's instructions, 4000 cells per well were seeded in a 96-well plate and then treated with test drugs for 72 h. Assays are performed by adding 10 µl of the CCK8 directly to culture wells, incubating for 1-4 h, and then recording the absorbance at 450 nm with a 96-well plate reader. After normalization by blank wells, the value of OD_450nm_ represents the number of viable cells.

### Quantitative RT-PCR

Quantification was performed using the CFX96 Touch Real-time PCR Detection system (Bio-Rad). Real-time quantitative reverse transcription PCR (qRT-PCR) was performed with S SYBR Green qPCR Master Mix (Bimake, USA) according to the manufacturer's instructions. GAPDH was used as an internal control. The sequence of primers was listed as follows: GAPDH forward primer: 5'- GGACTCATGACCACAGTCCA-3', reverse primer: 5'- TCAGCTCAGGGATGACCTTG-3'; DDX39 forward primer: 5'-TCCTCAAGAGAGCACACCAG-3', reverse primer: 5'-TGCTGGACCTCAGAAGGATG-3'. All experiments were performed in triplicate.

### Patients and microarray datasets

A total of 27 published BC high-throughput gene expression datasets containing following-up information was obtained from the Gene Expression Omnibus (GEO), Array Express, and The Cancer Genome Atlas (TCGA). GSE10885, GSE12093, GSE2034, GSE20624, GSE22226, GSE25066, GSE3143, GSE6532, GSE7390, GSE70947 and GSE7849 were collected from North America. Studies of GSE20685 and GSE3494 were conducted in Asia. All others including GSE11121, GSE12276, GSE1456, GSE21653, GSE22220, GSE24450, GSE42568, GSE4922, GSE53031, GSE58812 and NKI 30 were collected from Europe. TCGA1, TCGA2, and TCGA3 were obtained from TCGA (The Cancer Genome Atlas Program - National Cancer Institute). Detailed information on each dataset was summarized in Table S1.

The progression-free survival (PFS) period was defined as the time from initial surgery until tumor recurrence, including local relapse and distant metastasis. The overall survival (OS) time was calculated from the date of initial operation to the date when the patient was last seen. Kaplan-Meier survival plot was used to display the proportion of the population that were alive (OS) or progression-free (PFS) by the length of follow-up.

Participants were re-classified into four grades (1, 2, 3, and 4) according to expression levels of DDX39 at the percentile in the original dataset, which had been described in our previous publication 31. On the other hand, less than the value of the median was regarded as DDX39-low, and greater or equal to the median was DDX39-high.

### Bioinformatics and biostatistics analyses

Student t-test, one-way ANOVA, and non-parametric tests were used to test differences among subgroups for continuous data. The Pearson Chi-square and likelihood test was used for categorical data analyses. Kaplan-Meier analysis estimated the proportion of the population that were alive (OS) or disease-free (DFS) by the length of follow-up in months. Hazard ratios (HR) and 95% confidence intervals (CI) were calculated using Cox proportional hazards regression analysis. Two-sided P-values less than 0.05 were considered statistically significant. R and JMP statistical software were used for the above analysis unless otherwise noted.

Gene set enrichment analysis (GSEA) was applied to explore the relationship between DDX39 and cancer-related gene signatures and signaling pathways in the GSE1456 dataset. The detailed protocol of GSEA could be seen in our previous publications 32 and Broad Institute official website or from related references 33. Here, the number of permutation was set to 1000, and the phenotype label was DDX39-high versus DDX39-low.

## Results

### Inhibition of DDX39 causes growth retardation in BC cells

**Figure 1:**
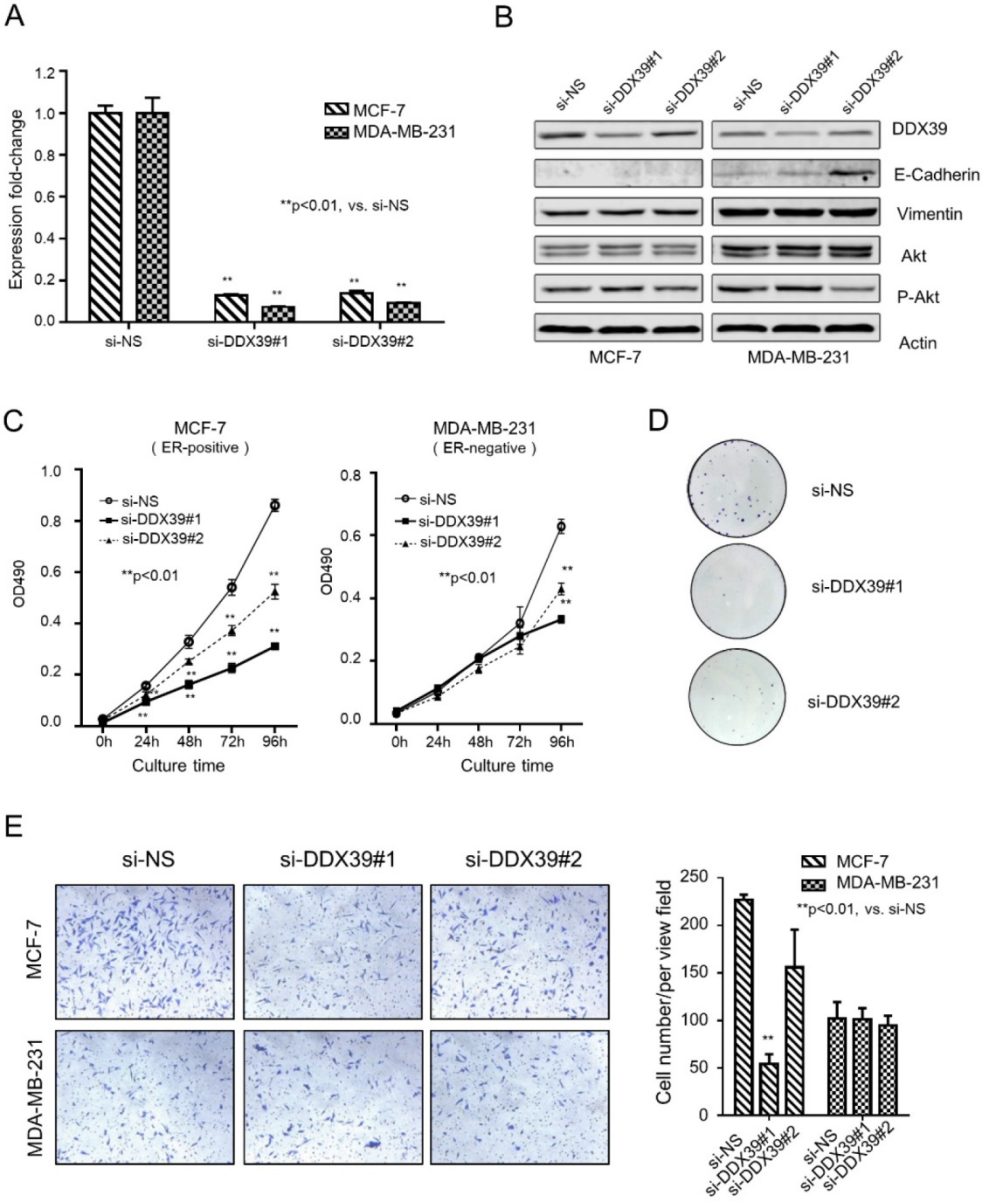
***DDX39* knockdown inhibited proliferation ability in MDA-MB-231 and MCF-7 cells.** A) qRT-PCR analyzed the expression of *DDX39*. B) The protein expression levels of *DDX39*, E-Cadherin, Vimentin, Akt, p-Akt, ERK and p-ERK in transfectants were visualized by western blot. C) The cell growth curve for these transfectants was determined by methylene blue staining. D) The colony formation of transfectants of MCF7 cells. E) An invasion chamber detected the invasive ability of transfectants.

An *in vitro* experiment demonstrated the relation between DDX39 and the development of BC. The expression of DDX39 was down-regulated by siRNA in ER-positive (MCF-7) and ER-negative (MDA-MB-231) BC cell lines (Fig. 1A and 1B). Results yielded from qRT-PCR and Western blot showed that si-DDX39 (#1 and #2) suppressed the mRNA and protein level of DDX39 significantly in both MCF-7 and MDA-MB-231 cells. In Fig. 1B, the Western blot showed that E-cadherin, an indicator of differentiation, dramatically increased in si-DDX39 (#2) transfectant in the MDA-MB-231 cell. Nevertheless, the E-cadherin could be barely seen in MCF-7 transfectants. The levels of Vimentin and Akt did not change significantly after DDX39 reduction, whereas p-AKT levels slightly decreased with inhibition of DDX39 (si-DDX39#2). Here, the N-Cadherin could not be visualized by western blot because of low expression in both cell lines.

As with inhibition of DDX39 by siRNAs, cell growth was significantly reduced after 24 hours in MCF-7 and 96 hours in MDA-MB-231 (Fig. 1C). The colony formation assay visualized the inhibition efficiency of si-DDX39 in MCF-7 cells (Fig. 1D). Experiments failed to show the colony formation results of MDA-MB-231 cells due to its weakness of adhesion ability. An invasion chamber measured the invasion capability of cancer cells. It was indicated that the capacity of invasion was slightly reduced by si-DDX39 in the MCF7 cell line, but not in the MDA-MB-231 cell line (Fig. 1E). The cell growth inhibition by DDX39 siRNAs also could be seen in another ER-positive BC cell line, ZR-75 (Fig. S1A, S1B, and S1C), which further confirmed the above findings.

These findings suggested that inhibition of DDX39 by siRNA could reduce the growth and invasion capability of cancer cells, especially in ER-positive BC cells (MCF-7 and ZR-75). Inhibition of DDX39 might affect the growth, but not the invasion, of MDA-MB-231 (ER-negative) cells.

### The expression of DDX39 associated with tumor growth and poor differentiation of BC

**Figure 2:**
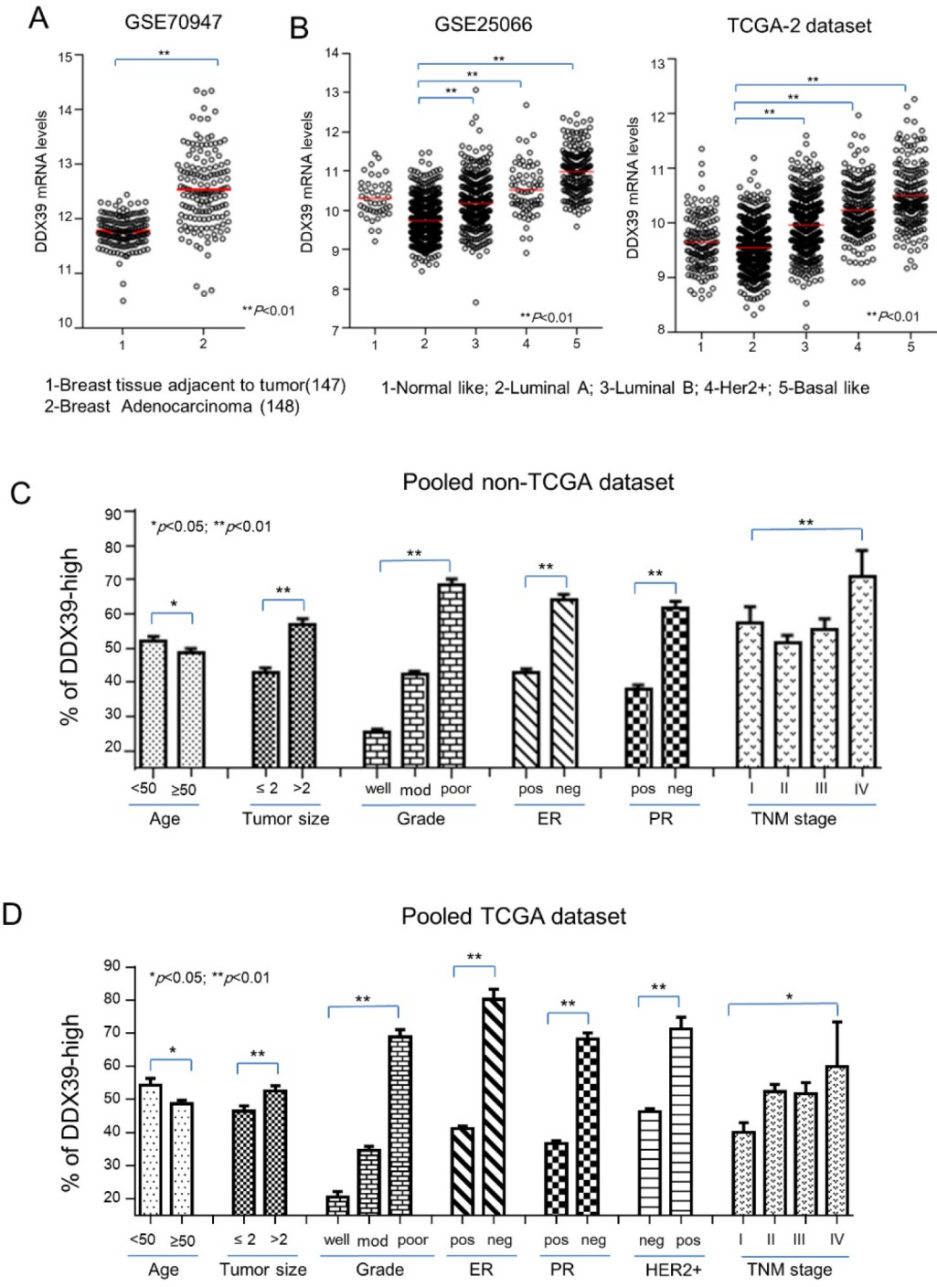
** Distribution of DDX39 expression on clinical features of BC.** The Clinical relevance of *DDX39* was evaluated on downloaded public gene expression datasets. A) Differential expression of *DDX39* between normal and BC tissues in the GSE70947 dataset. B) Expression of *DDX39* significantly associated with different molecular subtypes of BC in GSE25066 and TCGA-2 dataset. C/D) *DDX39* significantly related to factors including age, tumor size, gender, grade, ER, PR, Her2, and TNM stage in GEO/TCGA pooled dataset.

Based on the above findings, it implied that DDX39 might involve in the aggressiveness of BC. First, mRNA of DDX39 in breast adenocarcinoma tissues was significantly up-regulated in compared with adjacent normal breast tissue in the GSE70947 dataset (p<0.01) (Fig. 2A). In the GSE25066 and TCGA2 dataset, DDX39 expression was significantly higher in subtypes of Luminal B, Her2-positive, and basal-like BC, which were known as the molecular subtypes with the poorest prognosis (P<0.01) (Fig. 2B). The relation between the mRNA expression of DDX39 and the clinical features of BC were also analyzed in GEO and TCGA data sets. Results showed that the mRNA expression level of DDX39 was significantly associated with bigger tumor size, higher Elson grade and later TNM stage in pooled GEO and TCGA data set (p<0.05) (Fig. 2C, Fig. 2D and Table S2). These findings were compatible with an experimental analysis in Fig. 1. The investigation revealed that DDX39 would be a potential unfavorable biomarker for BC patients.

### Overexpression of DDX39 associates with poor survivability of ER-positive BC

**Figure 3:**
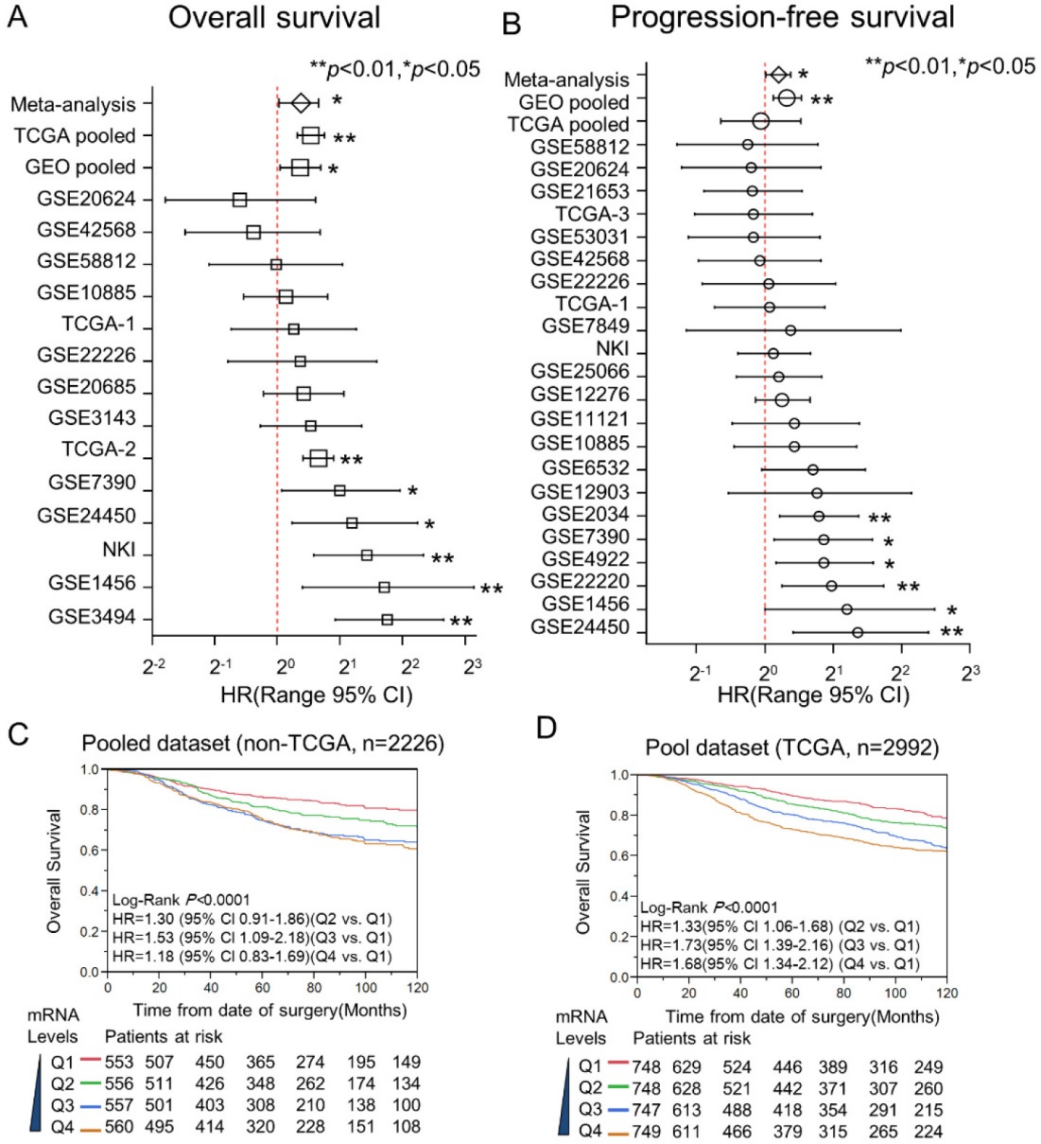
** Meta-analysis and pooled- analysis for *DDX39* and the outcome of BC.** The prognostic meaning of *DDX39* was determined in an individual and pooled manner. Meanwhile, the meta-analysis was used to adjust the heterogeneity of results yielded from each dataset. As for pooled data, they were merged after normalizing with quartile correction. A) Multivariate Cox analysis for OS in the individual and pooled dataset. B) HRs of *DDX39* for DFS were adjusted by pooled-analysis and meta-analysis. C) Kaplan-Meier analysis of *DDX39* impact the overall survival (OS) in pooled none TCGA dataset. The curves of red, green, blue, and brown represented Q1, Q2, Q3, and Q4 subgroups, respectively. The subgroup of Q1 was 0 to 25% percentile; Q2 was 25% to the median; Q3 was the median to 75% percentile, and Q4 was 75% percentile to the maximum. D) Expression of *DDX39* and OS in TCGA pooled dataset.

Here, Kaplan-Meier and Cox proportional hazard analysis for DDX39 were conducted on datasets with individual and pooled manner. Multivariate Cox analysis was performed to estimate the HR of DDX39 for OS and DFS in 22 GEO datasets, 1 NKI dataset, as well as 3 TCGA datasets (Fig. 3A and 3B). The participants of each dataset were recategorized into two subgroups (DDX39-low and DDX39-high) based on the DDX39 expression. The HRs of DDX39 (high vs. low) for OS reached statistical significance in 6 of 14 datasets. The statistical significance of DDX39 prognosticating poor DFS also could be seen in 6 of 22 datasets. There is no evidence showing that DDX39 is a favorable prognostic factor with statistical significance. The following pooled analysis and meta-analysis demonstrated that DDX39 significantly impacts poor OS and DFS of BC. The overall pooled analysis in GEO dataset indicated that the adjusted HR of DDX39 for OS and PFS was 1.29 (95% CI 1.03-1.62) and 1.30 (95% CI 1.13-1.50), respectively. An analysis in TCGA set validated that DDX39 significantly impact the overall survival of BC with adjusted HR was 1.56 (95% CI 1.25-1.69).

In the pooled GEO dataset, Kaplan-Meier curves visualized that the mRNA level of DDX39 was significantly associated with the inferior OS of BC (Fig. 3C). Here, all participants were stratified into four subgroups (Q1, Q2, Q3, and Q4) according to the expression levels of DDX39 in each dataset. As with the increase of DDX39 levels, the survivability of BC was getting poorer in a dose-dependent manner. This result could be validated in the pooled TCGA dataset (Fig. 3D).

### DDX39 specifically impacts the unfortunate outcome of ER-positive BC

**Figure 4:**
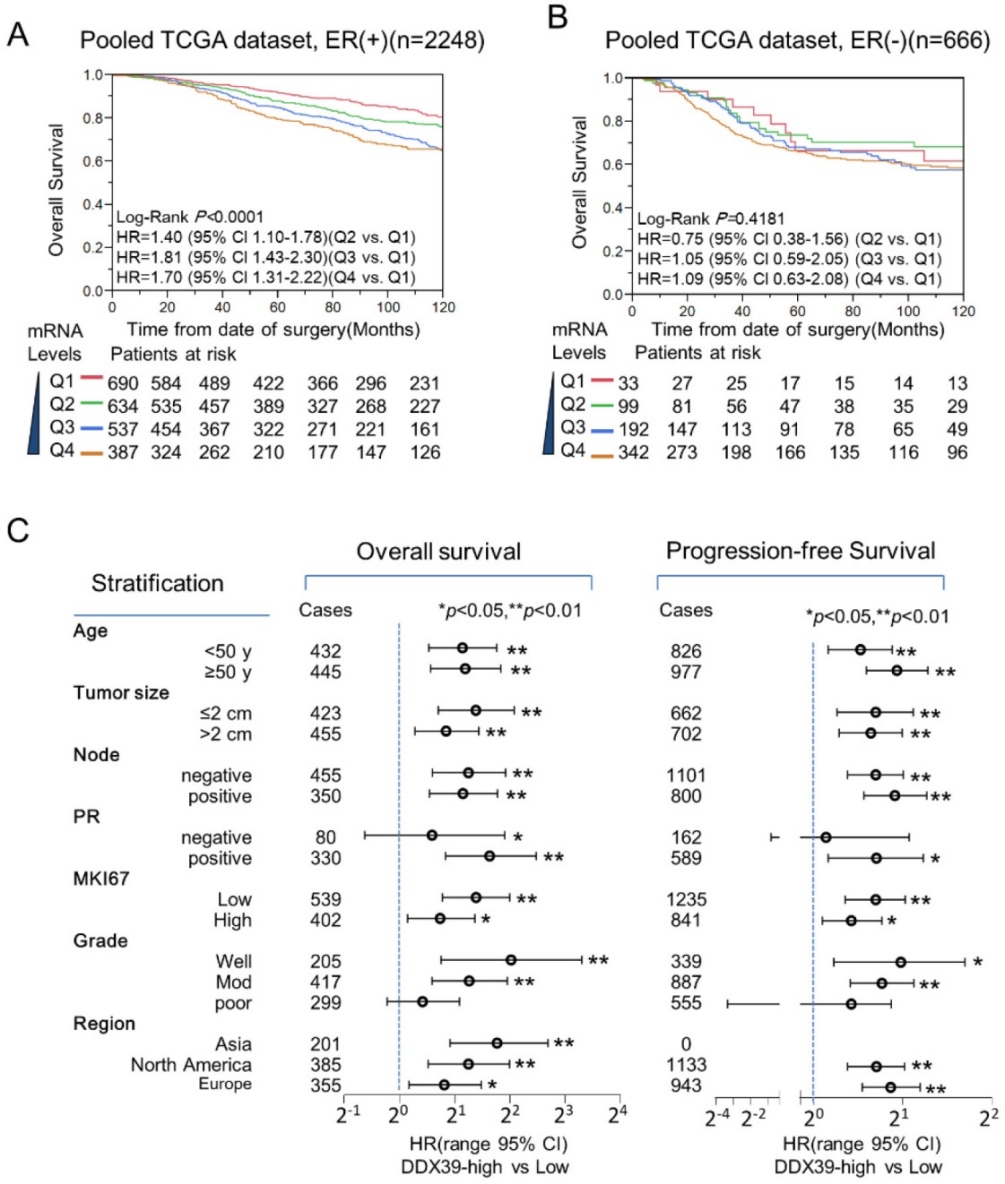
** Prognostic value of *DDX39* in ER-positive BC.** A) *DDX39* impacts poor overall survival (OS) of ER-positive BC in the GEO pooled dataset. B) *DDX39* predicts poor OS of ER-positive BC in TCGA pooled dataset. C) Stratification analysis for *DDX39* and OS/ PFS was conducted on pooled GEO dataset in ER-positive BC patients. The OS results were displayed on the left and PFS on the right. Participants were stratified by age, tumor size, lymph node, PR, MKI67, Elson grade and region. Asia subgroup includes GSE3494 and GSE20685 dataset; Europe subgroup contains GSE1456, GSE4922, GSE53031, GSE22220, GSE21653, GSE42568 and NKI dataset; and North America subgroup comprises GSE22226, GSE7390, GSE2034, GSE10885, GSE25066, GSE6532, GSE20624 and GSE7849 datasets. Cox analysis was conducted on each stratified subgroup.

Stratification analysis for the prognostic meaning of DDX39 in the pooled TCGA dataset revealed that DDX39 expression was associated with poorer OS in ER-positive significantly (Fig. 4A), but not ER-negative subtypes (Fig. 4B). In pooled GEO datasets, DDX39 expression was significantly associated with inferior OS in both ER-positive and ER-negative subtypes (data not shown). Nevertheless, a more remarkable dose-dependent fashion was observed in ER-positive subgroup than that in ER-negative subgroup. In Fig. 4C, further stratification analysis in pooled dataset indicated that DDX39 significantly associated with OS and PFS of ER-positive BC despite the status of age, tumor size, lymph node involvement, MKI67, and regions. The DDX39 associated with poor OS and DFS in PR-positive and lower Elson grade (1 and 2) BC, but not in PR-negative and Elson grade 3 patients. This finding seems confirmed experimental results (Fig. 1) that DDX39 promotes proliferation and invasion more significantly in ER-positive MCF7 cell than that in ER-negative MDA-MB-231 cell.

Meanwhile, the prognostic performance of DDX39 for ER-positive BC was further evaluated in NKI and TCGA datasets. In Fig. S2A and S2B, the DDX39 showed better predictive ability in ER-positive BC than that of the TNM stage in the NKI dataset. Furthermore, the prognostic capability of DDX39 was similar to multiple gene signatures (21-gene, 70-gene, and WR) and Elson grade, and was better than TNM stage in NKI dataset (Fig. S2C). Compatible prognostic performance of DDX39 to Elson grade and 21-gene signature also could be seen in TCGA dataset (Fig. S2D). Therefore, DDX39 is a promising prognostic indicator for those ER-positive BC patients.

### Reduction of DDX39 sensitizes chemotherapy in BC

**Figure 5:**
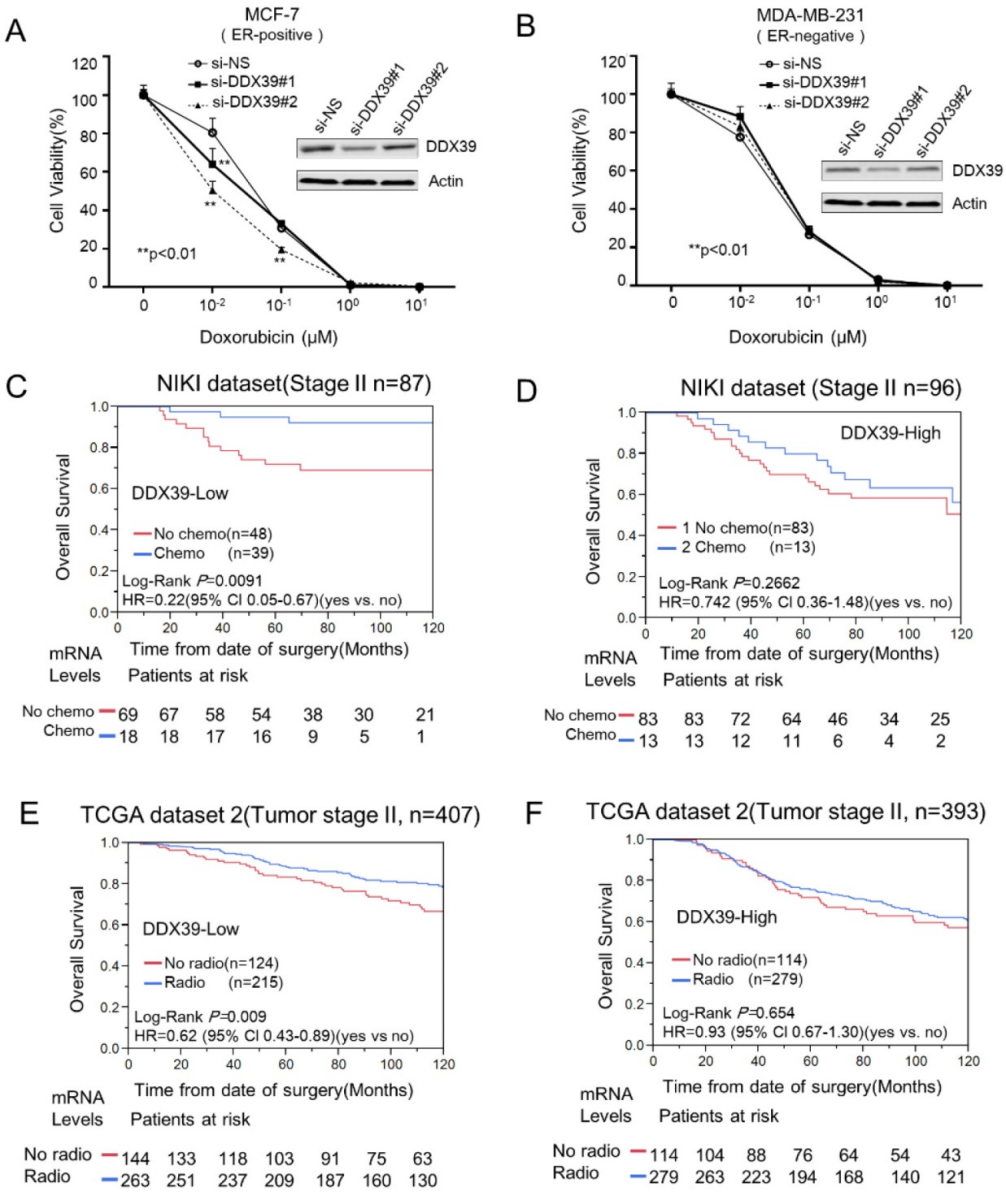
** The reduction of *DDX39* expression might enhance the cytotoxicity of doxorubicin in BCs.** About 4000 cells (MCF-7 or MDA-MB-231) were seeded on 96-cell plates and transfected with si-Neg and si-DDX39, respectively. Then, cells were treated with 0, 0.01, 0.1, 1, and 10 µM of doxorubicin for 72 h. Then, the cytotoxicity was measured by CCK8. The cytotoxicity curves for MCF-7 and MDA-MB-231 were displayed on A) and B), respectively. The chemotherapy efficacy between *DDX39*-high and *DDX39*-low was compared with stratification analysis on stage II BC patients from NIKI dataset. The Kaplan-Meier analysis results for *DDX39*-low and *DDX39*-high were shown on C) and D), respectively. In tumor stage II BC, Kaplan-Meier and multivariate Cox analyses were conducted to estimate the efficiency of radiotherapy for *DDX39*-low E) and *DDX39*-high F) subgroup in TCGA-2 dataset.

As well known, doxorubicin (also known as Adriamycin) is the most common therapeutic agents used on the therapeutic protocol in pre- or post-surgery BC treatment. Doxorubicin is known to augment the free-radical generation and lipid peroxidation process *in vitro* 34. We transfected si-DDX39 into MCF-7 and MDA-MB-231 cells to reduce their DDX39 expression. Then, we tested the cytotoxicity of doxorubicin on the above transfectants. Here, each cytotoxicity curve was normalized with a blank control. Results revealed that inhibition of DDX39 significantly enhanced the cytotoxicity to doxorubicin in MCF-7 (Fig. 5A), but not MDA-MB-231 cell (Fig. 5B). DDX39 siRNAs sensitized doxorubicin also could be observed in another ER-positive BC cell line, ZR-75 (Fig. S1D). The *in vitro* experiment implied that DDX39 expression might be related to chemoresistance to doxorubicin in ER-positive BC. The reduction of DDX39 might enhance drug sensitivity in chemotherapy for ER-positive BC patients.

To validate this finding, we further investigated the predictive significance of DDX39 in NKI and TCGA datasets. Generally, chemotherapy, radiotherapy and hormone selection were always determined by tumor size, lymph node involvement, metastasis, grade, ER/PR status, and other clinical factors. Therefore, these factors might be potential confounders in this evaluation. Here, only stage II BCs were selected for evaluating the efficiency of chemotherapy, radiotherapy, and hormone therapy in the NKI dataset. Therefore, confounders had been taken into consideration in this analysis. Analysis result indicated that chemotherapy could significantly reduce the risk of overall survival (HR=0.22; 95% CI 0.05-0.67) (Fig. 5C) in DDX39-low subgroup, but not in DDX39-high BC (Fig. 5D). Here, the efficiency of hormone therapy did not show differences between DDX39-high and DDX39-low subgroups (Data did not show). Nevertheless, the radiotherapy efficiency was not evaluated due to a lack of information in NKI dataset.

Meanwhile, we also examined the effects of therapies on tumor stage II and ER-positive BC in a TCGA dataset. Further stratification analysis indicated that radiotherapy could significantly reduce the risk of death in DDX39-low subgroup (HR= 0.62; 95% Cl 0.43-0.89) (Fig. 5E), but not in DDX39-high subgroup (HR=0.93; 95% Cl 0.67-1.30) (Fig. 5F). Meanwhile, the HR of chemotherapy for OS in DDX39-low subgroups (HR=0.84; 95% CI 0.46-1.45) was lower than that in DDX39-high BC (HR=1.34, 95% CI 0.76-2.21), but failed to show statistical significance in TCGA-2 dataset. Here, the efficacy of hormone therapy was not affected by DDX39 expression levels.

### The gene interaction network of DDX39 in BC

**Figure 6:**
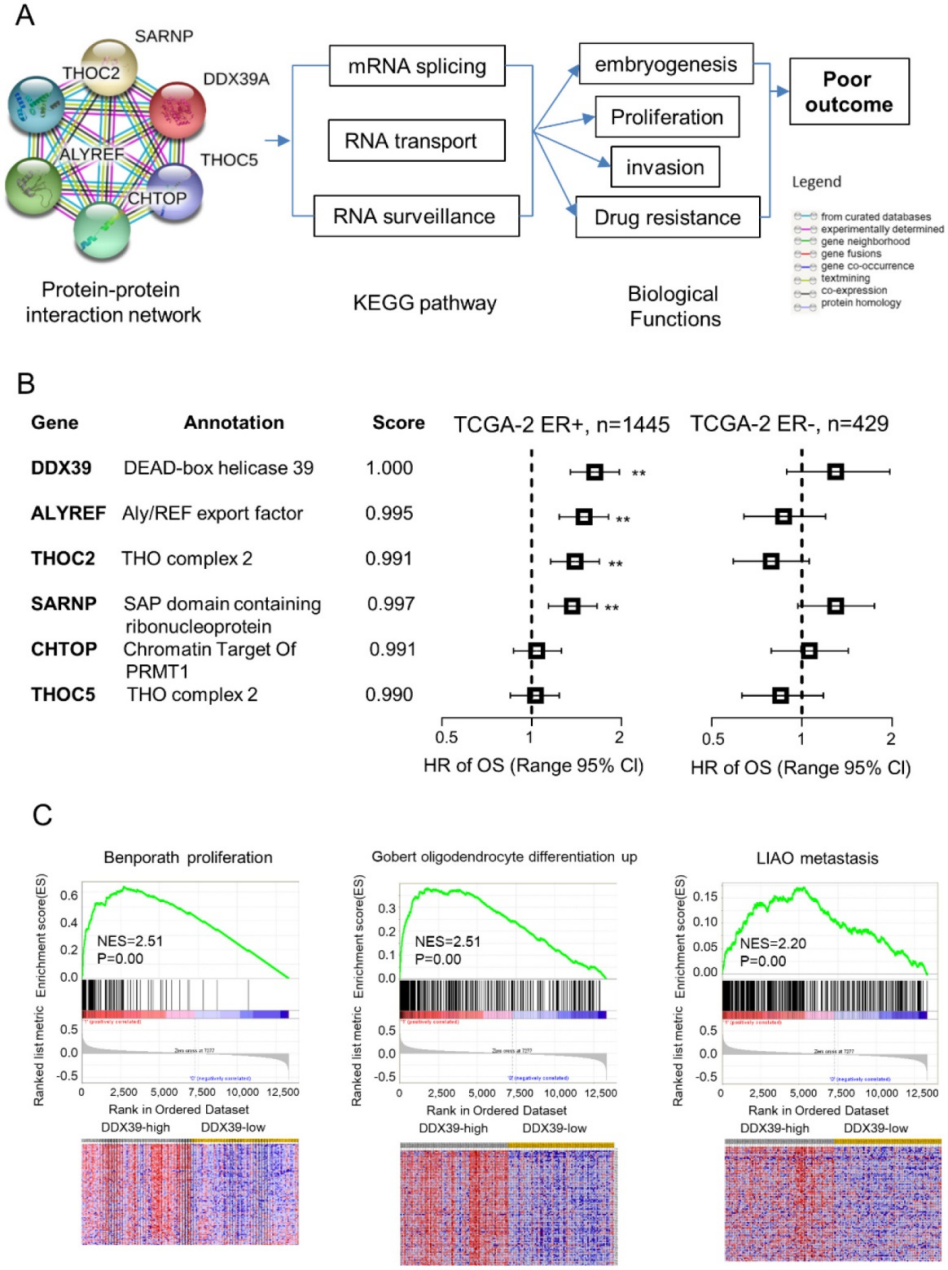
** Bioinformatics analysis for protein-protein interaction network of *DDX39*.** The investigation was conducted on STRING website. The interaction network of *DDX39* was determined from curated databases search, experimentally determined, gene neighborhood, gene fusions, co-occurrence, text mining, co-expression and protein homology. A) protein-protein interaction network of DDX39. B) Multivariate Cox analysis of *DDX39* and the related five genes were analyzed in the TCGA-2 dataset stratified by ER. C) Higher expression of DDX39 enriched signatures of Benporath proliferation, Gobert oligodendrocyte differentiation up, and Liao metastasis by GSEA in GSE1456 dataset. As for heatmap of GSEA, columns are cases which ranked by *DDX39* expression from high to low, and rows are genes of signature. Red represents the up-regulated gene, and blue is the down-regulated one.

To further understand the biological roles of DDX39 in ER-positive BC, we conducted the bioinformatics analysis for the protein-protein interaction network of DDX39 on STRING website. There have five genes coded proteins, THOC2, THOC5, ALYREF, SARNP and CHTOP were interacting with DDX39 by text mining (Fig. 6A). Further, KEGG pathway searching indicated that DDX39 was related to mRNA splicing, RNA transport, and RNA surveillance pathway. The prognostic meaning of these genes was further assessed in TCGA dataset. Multivariate Cox analysis revealed that THOC2, ALYREF, and SARNP were unfavorable prognostic factors with statistical significance in ER-positive BC, but not in ER-negative BC (Fig. 6B). THOC5 and CHTOP did not impact OS in either ER-positive or ER-negative BC.

Further gene set enrichment analysis (GSEA) was employed to explore the correlation between DDX39 levels and cancer-related gene signatures in GSE1456. The results suggested that high expression of DDX39 was significantly related to gene signatures, including Benporath proliferation, Gobert oligodendrocyte differentiation up, and Liao metastasis (Fig. 6C). Our findings suggested that DDX39 might involve in the regulation of mRNA splicing and RNA nuclear export to promote cancer cell proliferation and invasion in ER-positive BC.

## Discussion

DDX39 belongs to the DEAD-box RNA helicase family. It was reported to interact with ALY, CIP29 and FUS/TLS, involving regulation of transcription, splicing, and RNA export 1. The expression of DDX39 promotes cell growth and invasion ability, which had been reported in many research teams. DDX39 up-regulated in lung squamous cell carcinoma, and overexpression of DDX39 could stimulate colony formation of HeLa cells 5. Down-regulation of DDX39 inhibited cell migration and invasion in hepatocellular carcinoma cells, including Hep3B and Huh1 7. Our experiments demonstrated that inhibition of DDX39 by siRNA significantly increased the E-Cadherin and up-regulated p-AKT protein expression levels in MCF-7 and MDA-MB-231 cells (Fig. 1B). Meanwhile, inhibition of DDX39 caused the cancer cell growth retardation more significantly in ER-positive MCF7 cell than that in ER-negative MDA-MB-231 cell (Fig. 1C). However, siRNAs targeting DDX39 could reduce the invasion of MCF7, but not in MDA-MB-231 (Fig. 1E). Meanwhile, inhibition of cell growth by DDX39 siRNAs also could be seen in another ER-positive BC cell line (ZR-75) (Fig. S1), which further confirmed our finding from MCF-7 cell experiment. However, upregulation of DDX39 by expression plasmid could not change the colony formation and invasion ability in MCF-7 cells (data not shown). The reasons of why overexpression of DDX39 could promote the oncogenic property might be because overexpression of DDX39 in highly expressed BC cells might not trigger the dose-effect reaction anymore. Above findings suggested that DDX39 might a therapeutic target for BC with ER-positive.

The impact of DDX39 on ER-positive BC was validated in a large-scale population-based study. First, DDX39 mRNA overexpressed in the cancerous section of BC and positively correlated with advanced clinical features of BC (Fig. 2). Higher mRNA expression of DDX39 was associated with large tumor size, poor differentiation, and aggressive molecular subtypes. A meta-analysis demonstrated that DDX39 significantly associated with poor outcomes of BC (Fig. 3). Further stratification analysis revealed that DDX39 significantly prognosticates poor OS and DFS of ER-positive BC in a dose-dependent manner (Fig. 4A). However, the prognostic significance of DDX39 could not be seen in ER-negative BC (Fig. 4B). This finding consistent with our experimental result in Fig. 1. Moreover, DDX39 could serve as a prognostic biomarker for ER-positive BC, despite the clinical factors of age, tumor stage, lymph node involvement, and MKI67 status (Fig. 4C). Nevertheless, DDX39 could not be used prognostic biomarker for ER-positive BC with PR-negative or poor differentiation (Elson grade 3). The prognostic value of DDX39 could be observed in patients collected from Asia, Europe, as well as North America. Meanwhile, the prognostic performance of DDX39 in ER-positive BC was comparable to gene signatures of 70 genes, wound-response, and 21-gene (Oncotype DX), and better than TNM stage in NKI dataset (Fig. S2B). Similar prognostic efficiency between DDX39 and 21-gene signature for ER-positive BC also could be seen in TCGA dataset (Fig. S2C). Here, the biases and confounder had been taken into consideration. The heterogeneity of each dataset was corrected by pooled-analysis and meta-analysis. Therefore, the conclusion of DDX39 prognosticating poor outcome in ER-positive BC is reproducible and reliable.

The ER-positive BC accounts for about 60-70% of BC and has a relatively better long-term survival rate. Nowadays, there are serval classification platforms developed to identify the “poor-prognosis ER-positive BC” subtype for further chemotherapy and/ or radiotherapy 35. These multi-gene signatures are valuable for patients in avoiding overtreatment, prolonging the life-span, and improving life quality. Nevertheless, extensive investigation of mechanism and predictive meaning for these single gene is also essential. Here, the data showed that the high expression of DDX39 was also associated with resistance to chemotherapy and radiotherapy in ER-positive BC (Fig. 5). Inhibition of DDX39 could significantly enhance the sensitivity to doxorubicin in the MCF7 cell, but not MDA-MB-231 cell. This finding suggested that DDX39 might also serve as a predictive biomarker to predict the sensitivity to chemotherapy and radiotherapy for ER-positive BC. Meanwhile, it also might be a promising target for novel anti-cancer drug discovery.

The mechanism of DDX39 in the development of cancer and the involvement of drug resistance is still not clarified yet. Preliminary data demonstrated that DDX39 promotes HCC progression through the activating Wnt/β-catenin signaling pathway 7. Bioinformatic analysis suggested that proteins of THOC2, THOC5, ALYREF, SARNP and CHTOP genes might interact with DDX39 implicate in major biological processes involving alteration of RNA secondary structure such as mRNA splicing, RNA transport and RNA surveillance pathway (Fig. 6A). Based on the DDX39 protein-protein interaction network, we believed that it might be involved in embryogenesis and cellular growth. The GSEA result also confirmed that overexpression of DDX39 could enrich the gene signature of Benporath proliferation, Gobert oligodendrocyte differentiation up and Liao metastasis (Fig. 6C). DDX39 promoted cellular proliferation and invasion in ER-positive, rather than ER-negative BC cells (Fig. 1). THOC2, ALYREF and SARNP, DDX39 interacting proteins, played different prognostic roles between ER-positive and ER-negative BC patients (Fig. 6B). Inhibition of DDX39 only could enhance the drug sensitivity to chemotherapy in ER-positive BC (Fig. 5). Several other research teams also reported correlations between DDX39 and drug resistance. DDX39 was also up-regulated in malignant pleural mesothelioma cells and pancreatic cancer cells, which acquired gemcitabine resistance 36, 37. These findings suggested that DDX39 may play many critical roles in the development of estrogen driving BC. Nevertheless, further investigation needs to be carried out to figure out the essential mechanism of DDX39 in different ER status.

Nevertheless, the limitations of this study were taken into consideration. DDX39 plays different roles in drug sensitivity between ER-positive and ER-negative BC cells, which could partially explain that DDX39 had prognostic significance based on ER-status. The signaling pathways of cancer development need to be further investigated in ER-positive and ER-negative BC. The prognostic significance of DDX39 protein had not been assessed due to non-specific signal of DDX39 in immunohistochemistry analysis. In Fig. 5, chemotherapy could significantly reduce HR of OS (HR=0.22; 95% CI 0.05-0.67) in TNM stage II BC patients with DDX39-low, but not DDX39-high, in NKI dataset. This finding could not be exactly repeated in T stage II/ ER-positive BC in TCGA dataset, even different trends of chemotherapy could be observed between ER-positive and ER-negative BC. Here, we believe some missing factors, such as lymph node involvement and MKI67 status, might play as confounders and shift the HR of chemotherapy in both DDX39-low and DDX39-high subgroups in TCGA dataset.

The above findings suggested that DDX39 might be a novel prognostic and predictive biomarker for ER-positive BC. It could be used to predict outcomes and guide therapeutic protocol selection for BC with ER-positive status.

## Supplementary Material

Supplementary figures and tables.

## Abbreviations

DDX39: DEAD-box helicase 39
ER: estrogen receptor
GSEA: gene set enrichment analysis
HER2: Human epidermal growth factor receptor 2
HR: proportional hazard ratio
MKI-67: the marker of proliferation Ki-67
OS: overall survival
PFS: progression-free survival
PR: progesterone receptor
95% CI: 95% confidence interval

## References

[1] Sugiura T, Sakurai K, Nagano Y (2007). Intracellular characterization of DDX39, a novel growth-associated RNA helicase. Exp Cell Res.

[2] de la Cruz J, Kressler D, Linder P (1999). Unwinding RNA in Saccharomyces cerevisiae: DEAD-box proteins and related families. Trends Biochem Sci.

[3] Yoo HH, Chung IK (2011). Requirement of DDX39 DEAD box RNA helicase for genome integrity and telomere protection. Aging Cell.

[4] Rocak S, Linder P (2004). DEAD-box proteins: the driving forces behind RNA metabolism. Nat Rev Mol Cell Biol.

[5] Sugiura T, Nagano Y, Noguchi Y (2007). DDX39, upregulated in lung squamous cell cancer, displays RNA helicase activities and promotes cancer cell growth. Cancer Biol Ther.

[6] Kikuta K, Kubota D, Saito T, Orita H, Yoshida A, Tsuda H (2012). Clinical proteomics identified ATP-dependent RNA helicase DDX39 as a novel biomarker to predict poor prognosis of patients with gastrointestinal stromal tumor. J Proteomics.

[7] Zhang T, Ma Z, Liu L, Sun J, Tang H, Zhang B (2018). DDX39 promotes hepatocellular carcinoma growth and metastasis through activating Wnt/beta-catenin pathway. Cell Death Dis.

[8] Kato M, Wei M, Yamano S, Kakehashi A, Tamada S, Nakatani T (2012). DDX39 acts as a suppressor of invasion for bladder cancer. Cancer Sci.

[9] Samson M, Porter N, Orekoya O, Hebert JR, Adams SA, Bennett CL (2016). Progestin and breast cancer risk: a systematic review. Breast Cancer Res Treat.

[10] Chand AR, Ziauddin MF, Tang SC (2017). Can Locoregionally Recurrent Breast Cancer Be Cured?. Clin Breast Cancer.

[11] Sosa MS, Bragado P, Aguirre-Ghiso JA (2014). Mechanisms of disseminated cancer cell dormancy: an awakening field. Nat Rev Cancer.

[12] Pantel K, Brakenhoff RH (2004). Dissecting the metastatic cascade. Nat Rev Cancer.

[13] Sorlie T, Wang Y, Xiao C, Johnsen H, Naume B, Samaha RR (2006). Distinct molecular mechanisms underlying clinically relevant subtypes of breast cancer: gene expression analyses across three different platforms. BMC Genomics.

[14] Goldhirsch A, Wood WC, Coates AS, Gelber RD, Thurlimann B, Senn HJ (2011). Strategies for subtypes-dealing with the diversity of breast cancer: highlights of the St. Gallen International Expert Consensus on the Primary Therapy of Early Breast Cancer 2011. Ann Oncol.

[15] Carey LA, Perou CM, Livasy CA, Dressler LG, Cowan D, Conway K (2006). Race, breast cancer subtypes, and survival in the Carolina Breast Cancer Study. JAMA.

[16] Pritchard KI (2013). Endocrine therapy: is the first generation of targeted drugs the last?. J Intern Med.

[17] Osborne CK (1998). Tamoxifen in the treatment of breast cancer. N Engl J Med.

[18] Gradishar W, Salerno KE (2016). NCCN Guidelines Update: Breast Cancer. J Natl Compr Canc Netw.

[19] Li JJ, Shao ZM (2016). Endocrine therapy as adjuvant or neoadjuvant therapy for breast cancer: selecting the best agents, the timing and duration of treatment. Chin Clin Oncol.

[20] Cheng L, Swartz MD, Zhao H, Kapadia AS, Lai D, Rowan PJ (2012). Hazard of recurrence among women after primary breast cancer treatment-a 10-year follow-up using data from SEER-Medicare. Cancer Epidemiol Biomarkers Prev.

[21] Metzger-Filho O, Sun Z, Viale G, Price KN, Crivellari D, Snyder RD (2013). Patterns of Recurrence and outcome according to breast cancer subtypes in lymph node-negative disease: results from international breast cancer study group trials VIII and IX. J Clin Oncol.

[22] Ribelles N, Perez-Villa L, Jerez JM, Pajares B, Vicioso L, Jimenez B (2013). Pattern of recurrence of early breast cancer is different according to intrinsic subtype and proliferation index. Breast Cancer Res.

[23] Sgroi DC, Sestak I, Cuzick J, Zhang Y, Schnabel CA, Schroeder B (2013). Prediction of late distant recurrence in patients with oestrogen-receptor-positive breast cancer: a prospective comparison of the breast-cancer index (BCI) assay, 21-gene recurrence score, and IHC4 in the TransATAC study population. Lancet Oncol.

[24] Sestak I, Cuzick J, Dowsett M, Lopez-Knowles E, Filipits M, Dubsky P (2015). Prediction of late distant recurrence after 5 years of endocrine treatment: a combined analysis of patients from the Austrian breast and colorectal cancer study group 8 and arimidex, tamoxifen alone or in combination randomized trials using the PAM50 risk of recurrence score. J Clin Oncol.

[25] Harris LN, Ismaila N, McShane LM, Hayes DF (2016). Use of Biomarkers to Guide Decisions on Adjuvant Systemic Therapy for Women With Early-Stage Invasive Breast Cancer: American Society of Clinical Oncology Clinical Practice Guideline Summary. J Oncol Pract.

[26] Paik S, Shak S, Tang G, Kim C, Baker J, Cronin M (2004). A multigene assay to predict recurrence of tamoxifen-treated, node-negative breast cancer. N Engl J Med.

[27] Sestak I, Dowsett M, Zabaglo L, Lopez-Knowles E, Ferree S, Cowens JW (2013). Factors predicting late recurrence for estrogen receptor-positive breast cancer. J Natl Cancer Inst.

[28] Buus R, Sestak I, Kronenwett R, Denkert C, Dubsky P, Krappmann K (2016). Comparison of EndoPredict and EPclin With Oncotype DX Recurrence Score for Prediction of Risk of Distant Recurrence After Endocrine Therapy.

[29] Liu X, Lai L, Wang X, Xue L, Leora S, Wu J (2011). Ribonucleotide reductase small subunit M2B prognoses better survival in colorectal cancer. Cancer Res.

[30] van de Vijver MJ, He YD, van't Veer LJ, Dai H, Hart AA, Voskuil DW (2002). A gene-expression signature as a predictor of survival in breast cancer. N Engl J Med.

[31] Zhang H, Liu X, Warden CD, Huang Y, Loera S, Xue L (2014). Prognostic and therapeutic significance of ribonucleotide reductase small subunit M2 in estrogen-negative breast cancers. BMC Cancer.

[32] Zhang L, Chen Z, Xue D, Zhang Q, Liu X, Luh F (2016). Prognostic and therapeutic value of mitochondrial serine hydroxyl-methyltransferase 2 as a breast cancer biomarker. Oncology reports.

[33] Subramanian A, Tamayo P, Mootha VK, Mukherjee S, Ebert BL, Gillette MA (2005). Gene set enrichment analysis: a knowledge-based approach for interpreting genome-wide expression profiles. Proc Natl Acad Sci U S A.

[34] Krishnan N, Dickman MB, Becker DF (2008). Proline modulates the intracellular redox environment and protects mammalian cells against oxidative stress. Free Radic Biol Med.

[35] Lonning PE (2012). Poor-prognosis estrogen receptor- positive disease: present and future clinical solutions. Therapeutic advances in medical oncology.

[36] Kuramitsu Y, Tominaga W, Baron B, Tokuda K, Wang Y, Kitagawa T (2013). Up-regulation of DDX39 in human malignant pleural mesothelioma cell lines compared to normal pleural mesothelial cells. Anticancer Res.

[37] Kuramitsu Y, Suenaga S, Wang Y, Tokuda K, Kitagawa T, Tanaka T (2013). Up-regulation of DDX39 in human pancreatic cancer cells with acquired gemcitabine resistance compared to gemcitabine-sensitive parental cells. Anticancer Res.

