# Supplementary material for: DEAD-box RNA Helicase 39 Promotes Invasiveness and Chemoresistance of ER-positive Breast Cancer
Source: J Cancer. 2020 Jan 20;11(7):1846–58. doi: 10.7150/jca.37247 (PMC7052869; doi:10.7150/jca.37247)
Supplement: Supplementary file 1 — Supplementary figures and tables. [file jcav11p1846s1.pdf]

# Supplementary Figure Legend

**Figure S1. DDX39 siRNAs reduced cell proliferation and sensitized the cytotoxicity to doxorubicin in ZR-75 cells.** A) inhibition of DDX39 mRNA by siRNAs in ZR-75 cells. B) Western analysis for expression of DDX39, Vimentin, Akt, p-Akt. C) Effects of DDX39 siRNAs in cell growth of ZR-75. D) Enhancing cytotoxicity to doxorubicin by DDX39 siRNAs.

**Figure S2. Comparing the prognostic performance of DDX39 with gene signatures in ER-positive BC.** A) Kaplan-Meier analysis for DDX39 and OS of ER-positive BC was conducted in the NKI dataset. B) The TNM stage and OS of ER-positive BC were visualized in the NKI dataset. C) The prognostic performances of *DDX39* gene signatures and the TNM stage were compared in the NKI dataset. Here, 70-gene is the 70-gene signature; WR is the wound-response gene signature; 21-gene is the 21-gene recurrence score (Oncotype DX). D) The validation of prognostic performances of *DDX39* expression, age, grade, and the 21-gene recurrence score was performed in the TCGA2 dataset.

Figure S1

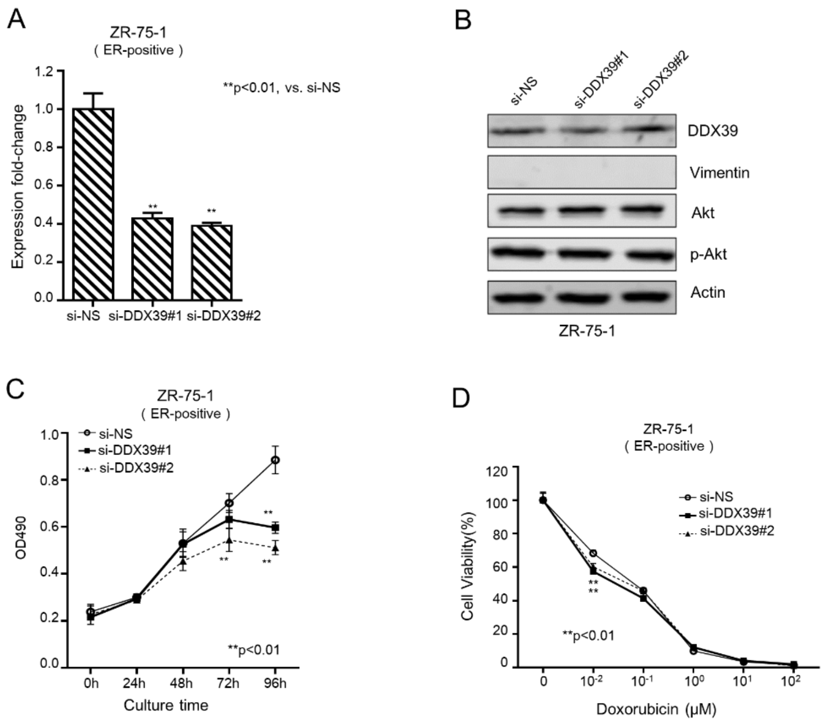

A
ZR-75-1
( ER-positive )
**p<0.01, vs. si-NS
Expression fold-change
si-NS
si-DDX39#1
si-DDX39#2
B
si-NS
si-DDX39#1
si-DDX39#2
DDX39
Vimentin
Akt
p-Akt
Actin
ZR-75-1
C
ZR-75-1
( ER-positive )
si-NS
si-DDX39#1
si-DDX39#2
OD490
**p<0.01
0h
24h
48h
72h
96h
Culture time
D
ZR-75-1
( ER-positive )
si-NS
si-DDX39#1
si-DDX39#2
Cell Viability(%)
**p<0.01
Doxorubicin (μM)

Figure S2

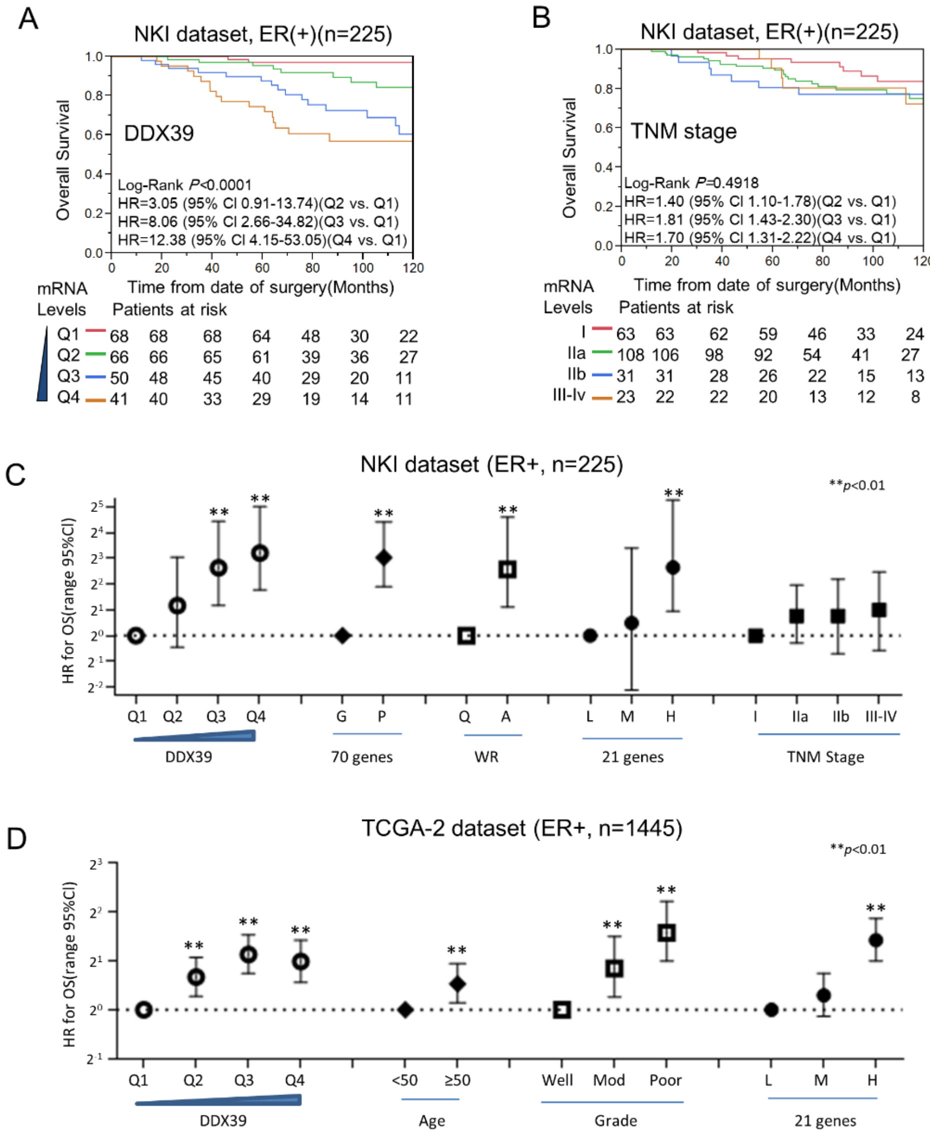


**Table S1. Summary of worldwide gene expression datasets of breast cancer**

| Dataset | No. of | Patients w/ | Platforms* | Country | Molecular | Information about Therapies | OS | PFS |
|---|---|---|---|---|---|---|---|---|

| | Patients | outcome | | | subtype | Chemo | Radio | Hormone | | |
|---|---|---|---|---|---|---|---|---|---|---|
| GSE10885 | 244 | 154 | GPL885,887 | USA | N/A | N/A | N/A | N/A | Y | Y |
| GSE11121 | 200 | 200 | GPL96 | Germany | N/A | N/A | N/A | N/A | N/A | Y |
| GSE12093 | 136 | 136 | GPL96 | USA | N/A | N/A | N/A | N/A | N/A | Y |
| GSE12276 | 204 | 204 | GPL570 | Netherlands | N/A | N/A | N/A | N/A | N/A | Y |
| GSE1456 | 159 | 159 | GPL96,97,570 | Sweden | Y | N/A | N/A | N/A | Y | Y |
| GSE2034 | 286 | 286 | GPL96 | USA | N/A | N/A | N/A | N/A | N/A | Y |
| GSE20624 | 510 | 176 | GPL885, 887 | USA | Y | N/A | N/A | N/A | Y | Y |
| GSE20685 | 327 | 327 | GPL570 | Taiwan | N/A | N/A | N/A | N/A | Y | Y |
| GSE21653 | 266 | 266 | GPL570 | France | Y | N/A | N/A | N/A | N/A | Y |
| GSE22220 | 216 | 216 | GPL6098 | UK | N/A | N/A | N/A | N/A | N/A | Y |
| GSE22226 | 150 | 129 | GPL1708,4133 | USA | Y | Y | N/A | Y | Y | Y |
| GSE24450 | 183 | 183 | GPL6947 | Finland | N/A | N/A | N/A | N/A | Y | Y |
| GSE25066 | 508 | 508 | GLP96 | USA | N/A | N/A | N/A | N/A | N/A | Y |
| GSE3143 | 158 | 158 | GPL8300 | USA | N/A | N/A | N/A | N/A | Y | N/A |
| GSE3494 | 502 | 251 | GPL96,97 | Singapore | N/A | N/A | N/A | N/A | Y | N/A |
| GSE42568 | 123 | 105 | GPL570 | Ireland | N/A | N/A | N/A | N/A | Y | Y |
| GSE4922 | 578 | 289 | GPL96,97 | Singapore | N/A | N/A | N/A | N/A | N/A | Y |
| GSE53031 | 167 | 167 | GPL13667 | Belgium | Y | N/A | N/A | N/A | N/A | Y |
| GSE58812 | 107 | 107 | GPL570 | France | N/A | N/A | N/A | N/A | Y | Y |
| GSE6532 | 741 | 244 | GPL96,97,570 | Canada | N/A | Y | N/A | N/A | N/A | Y |
| GSE7390 | 198 | 198 | GPL96 | Canada | N/A | N/A | N/A | N/A | Y | Y |
| GSE7849 | 78 | 78 | GPL8300 | USA | N/A | Y | N/A | Y | N/A | Y |
| NKI# | 295 | 295 | Agilent 25K chip | Netherlands | Y | Y | N/A | Y | Y | Y |
| TCGA-1 | 526 | 526 | N/A | N/A | N/A | N/A | N/A | N/A | Y | Y |
| TCGA-2 | 1904 | 1904 | N/A | N/A | Y | Y | Y | Y | Y | N/A |
| TCGA-3 | 564 | 564 | N/A | N/A | N/A | Y | Y | N/A | Y | Y |

* Platforms: GPL96:[HG-U133A] Affymetrix Human Genome U133A Array; GPL97: [HG-U133B] Affymetrix Human Genome U133B Array; GPL570: [HG-U133_Plus_2] Affymetrix Human Genome U133 Plus 2.0 Array; GPL13667: [HG-U219] Affymetrix Human Genome U219 Array; GPL8269: Agilent UNC PerouLab 244K Custom Human Array version 5; GPL1708: Agilent-012391 Whole Human Genome Oligo Microarray G4112A ; GPL4133: Agilent-014850 Whole Human Genome Microarray 4x44K G4112F; GPL885: Agilent-011521 Human 1A Microarray G4110A; GPL887: Agilent-012097 Human 1A Microarray (V2) G4110B; GPL6947: Illumina HumanHT-12 V3.0 expression beadchip; GPL6098:Illumina humanRef-8 v1.0 expression beadchip; GPL6947: Illumina HumanHT-12 V3.0 expression beadchip; GPL6098:Illumina humanRef-8 v1.0 expression beadchip; GPL8300:[HG_U95Av2] Affymetrix Human Genome U95 Version 2 Array.

# Table S2. Demographic distribution of DDX39

| | Pooled GEO datasets | | | Pooled TCGA dataset | | |
|---|---|---|---|---|---|---|
| | Low(%*) | High(%*) | *p* value† | Low(%*) | High(%*) | *p* value† |
| **Age** | | | | | | |
| <50 | 776(47.8) | 846(52.2) | | 322(45.7) | 383(54.3) | |
| ≥50 | 936(51.2) | 891(48.8) | 0.047 | 1174(51.3) | 1115(48.7) | 0.009 |
| **Grade** | | | | | | |
| 1=Well | 372(74.7) | 126(25.3) | | 131(79.4) | 34(20.6) | |
| 2=Mod | 836(57.8) | 611(42.2) | | 485(65.5) | 256(34.5) | |
| 3=Poor | 419(31.7) | 902(68.3) | <0.001 | 289(31.2) | 637(68.8) | <0.001 |
| **ER** | | | | | | |
| Negative | 448(36.1) | 794(63.9) | | 132(19.8) | 534(80.2) | |
| Positive | 1305(56.9) | 988(43.1) | <0.001 | 1324(58.9) | 925(41.1) | <0.001 |
| **PR** | | | | | | |
| Negative | 310(38.5) | 495(61.5) | | 393(31.8) | 843(68.2) | |
| Positive | 518(62.0) | 318(38.0) | <0.001 | 1080(63.3) | 626(36.7) | <0.001 |
| **HER2+** | | | | | | |
| Negative | 631(50.3) | 624(49.7) | | 1198(53.8) | 1029(46.2) | |
| Positive | 73(44.2) | 92(55.8) | 0.145 | 115(28.8) | 285(71.2) | <0.001 |
| **Tumor size** | | | | | | |
| ≤2 | 652(56.9) | 493(43.1) | | 441(53.6) | 382(46.4) | |
| >2 | 561(43.0) | 744(57.0) | <0.001 | 505(47.5) | 558(52.5) | 0.009 |
| **Lymph node** | | | | | | |
| Negative | 989(50.2) | 982(49.8) | | 268(52.0) | 247(48.0) | |
| Positive | 645(48.9) | 674(51.1) | 0.473 | 267(48.0) | 289(52.0) | 0.412 |
| **TNM stage** | | | | | | |
| I | 66(42.6) | 89(57.4) | | 108(60.0) | 72(40.0) | |
| II | 259(48.4) | 276(51.6) | | 294(47.6) | 324(52.4) | |
| III | 120(44.8) | 148(55.2) | | 120(48.2) | 129(51.8) | |
| IV | 24(29.3) | 58(70.3) | 0.011 | 8(40.0) | 12(60.0) | 0.045 |
| **Molecular subtype** | | | | | | |
| Luminal A | 394(75.2) | 130(24.8) | | 522(76.9) | 157(23.1) | |
| Luminal B | 125(37.2) | 211(62.8) | | 194(42.1) | 267(57.9) | |
| HER2 + | 89(44.5) | 111(55.5) | | 38(17.3) | 182(82.7) | |
| Basal | 197(31.6) | 426(68.4) | | 19(9.6) | 180(90.4) | |
| Normal | 143(68.7) | 65(31.3) | <0.001 | 95(67.9) | 45(32.1) | <0.001 |

Note: In the GEO dataset, there are 3449, 3266, 3535, 1642, 1420, 2450, 3290, 1916, 1040 in Age, Grade, ER, PR, HER2+, Tumor size, Lymph node status, Molecular subtype, Pathological TNM stage. In the TCGA dataset, there are 2994, 1832, 2915, 2942, 2627, 1886, 1071, 1898, 1067 in Age, Grade, ER, PR, HER2+, Tumor size, Lymph node status, Molecular subtype, Pathological TNM stage.

* % represents the positive rate of DDX39-High it equal to $N_{High}/(N_{High}+N_{Low})\times 100\%$.

† *p* values were based on the Pearson Chi-square test.

Original picture of Western blot

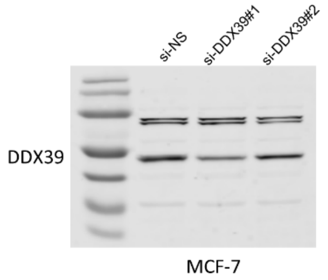
si-NS
si-DDX39#1
si-DDX39#2
DDX39
MCF-7

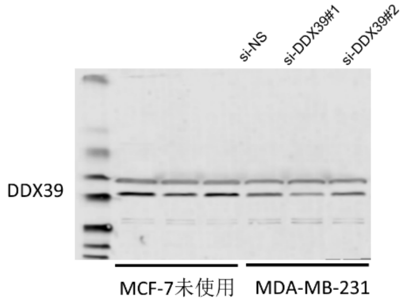
si-NS
si-DDX39#1
si-DDX39#2
DDX39
MCF-7未使用
MDA-MB-231

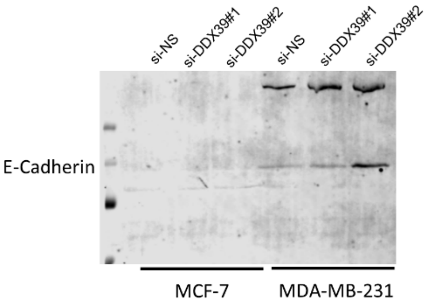
si-NS
si-DDX39#1
si-DDX39#2
si-NS
si-DDX39#1
si-DDX39#2
E-Cadherin
MCF-7
MDA-MB-231

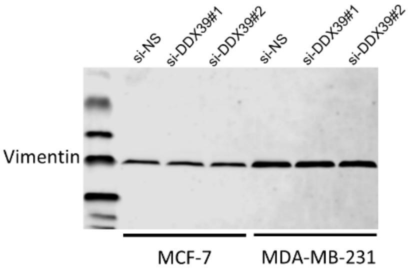
si-NS
si-DDX39#1
si-DDX39#2
si-NS
si-DDX39#1
si-DDX39#2
Vimentin
MCF-7
MDA-MB-231

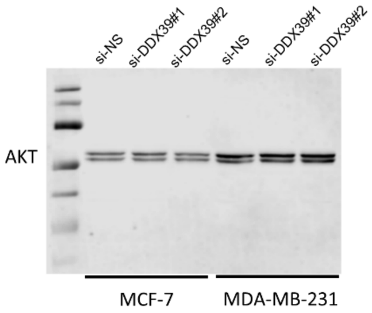
si-NS
si-DDX39#1
si-DDX39#2
si-NS
si-DDX39#1
si-DDX39#2
AKT
MCF-7
MDA-MB-231

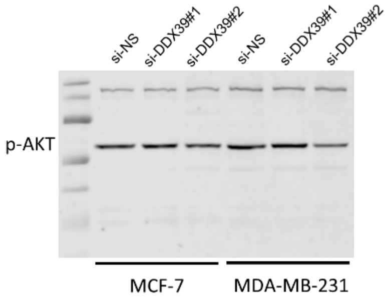
si-NS
si-DDX39#1
si-DDX39#2
si-NS
si-DDX39#1
si-DDX39#2
p-AKT
MCF-7
MDA-MB-231

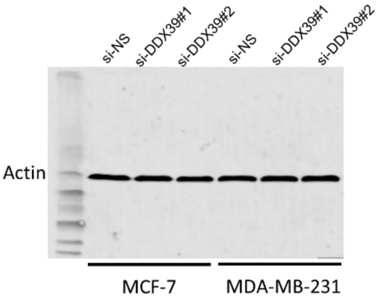
si-NS
si-DDX39#1
si-DDX39#2
si-NS
si-DDX39#1
si-DDX39#2
Actin
MCF-7
MDA-MB-231

Original picture of colony formation

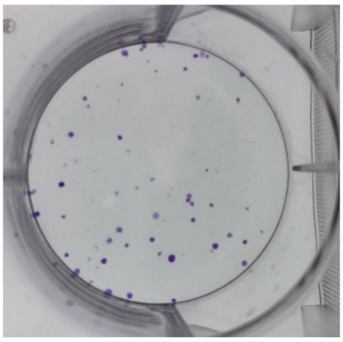

si-neg

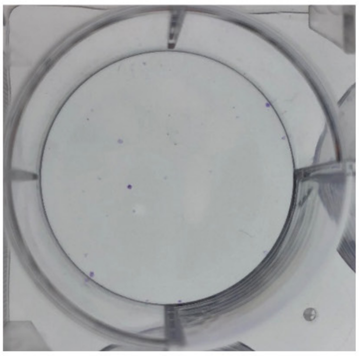

si-DDX39#1

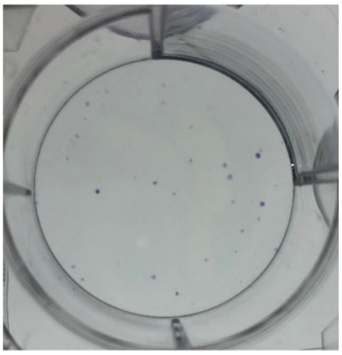

si-DDX39#2
